# The potato cyst nematode effector RHA1B is a ubiquitin ligase and uses two distinct mechanisms to suppress plant immune signaling

**DOI:** 10.1371/journal.ppat.1007720

**Published:** 2019-04-12

**Authors:** Joanna Kud, Wenjie Wang, Rachel Gross, Youhong Fan, Li Huang, Yulin Yuan, Amanda Gray, Aida Duarte, Joseph C. Kuhl, Allan Caplan, Aska Goverse, Yongsheng Liu, Louise-Marie Dandurand, Fangming Xiao

**Affiliations:** 1 Department of Plant Sciences, University of Idaho, Moscow, ID, United States of America; 2 School of Food Science, Hefei University of Technology, Hefei, China; 3 Department of Entomology, Plant Pathology and Nematology, University of Idaho, Moscow, ID, United States of America; 4 Laboratory of Nematology, Department of Plant Sciences, Wageningen University & Research, Wageningen, The Netherlands; 5 School of Horticulture, Anhui Agricultural University, Hefei, China; 6 Ministry of Education Key Laboratory for Bio-resource and Eco-environment, College of Life Science, State Key Laboratory of Hydraulics and Mountain River Engineering, Sichuan University, Chengdu, China; University of California, Riverside, UNITED STATES

## Abstract

Plant pathogens, such as bacteria, fungi, oomycetes and nematodes, rely on wide range of virulent effectors delivered into host cells to suppress plant immunity. Although phytobacterial effectors have been intensively investigated, little is known about the function of effectors of plant-parasitic nematodes, such as *Globodera pallida*, a cyst nematode responsible for vast losses in the potato and tomato industries. Here, we demonstrate using *in vivo* and *in vitro* ubiquitination assays the potato cyst nematode (*Globodera pallida*) effector RHA1B is an E3 ubiquitin ligase that employs multiple host plant E2 ubiquitin conjugation enzymes to catalyze ubiquitination. RHA1B was able to suppress effector-triggered immunity (ETI), as manifested by suppression of hypersensitive response (HR) mediated by a broad range of nucleotide-binding leucine-rich repeat (NB-LRR) immune receptors, presumably via E3-dependent degradation of the NB-LRR receptors. RHA1B also blocked the flg22-triggered expression of *Acre31* and *WRKY22*, marker genes of pathogen‐associated molecular pattern (PAMP)‐triggered immunity (PTI), but this did not require the E3 activity of RHA1B. Moreover, transgenic potato overexpressing the *RHA1B* transgene exhibited enhanced susceptibility to *G*. *pallida*. Thus, our data suggest RHA1B facilitates nematode parasitism not only by triggering degradation of NB-LRR immune receptors to block ETI signaling but also by suppressing PTI signaling via an as yet unknown E3-independent mechanism.

## Author summary

*Globodera pallida* is a plant-parasitic cyst nematode that causes vast losses in economically important crops such as potato and tomato. To successfully parasitize host plants, *G*. *pallida* produces proteins called effectors to overcome plant defenses. Here, we report identification of a novel *G*. *pallida* effector RHA1B as an E3 ubiquitin ligase, which is responsible for ubiquitin-proteasome-mediated protein degradation in general. We found that RHA1B can suppress plant defense signaling via both E3-dependent and -independent manners. In particular, it promotes degradation of a broad range of NB-LRR immune receptors. In addition, expression of RHA1B in potato plants made the plants more susceptible to *G*. *pallida* infection, indicating that RHA1B acts as an effector that aids parasitism. Overall, we found RHA1B as the first effector with ubiquitin ligase activity identified from eukaryotic pathogen infecting plants or animals. Our data suggest nematode uses RHA1B as a powerful weapon to manipulate host cellular signaling pathways, thereby interfering with plant immunity for successful parasitism.

## Introduction

*Globodera pallida*, a plant-parasitic cyst nematode, is a global threat to agronomically important crops such as potato and tomato. This sedentary plant endoparasite penetrates plant root systems to reach the inner cortex where it establishes a permanent feeding site, a multi-nucleate structure termed syncytium [1]. The transformation of plant root cells into syncytia by cyst nematodes is mediated by effectors produced in the nematode pharyngeal glands. It is generally believed that nematode effectors must manipulate various host physiological processes, particularly suppressing the plant defense responses, via largely unknown mechanisms, in order to facilitate the formation and maintenance of syncytia [1].

The first layer of plant defense is governed by membrane-associated pattern recognition receptors (PRRs) that recognize pathogen associated molecular patterns (PAMPs) and/or endogenous damage-associated molecular patterns (DAMPs). This is termed PAMP-triggered immunity (PTI) and, to overcome PTI in host plants, pathogens use the secreted effectors to suppress PTI [2]. In turn, to counteract the interference of PTI by pathogen effectors, resistant plants have evolved a second layer of defense termed effector-triggered immunity (ETI), which is mediated by recognition of specific effectors by cytoplasmic NB-LRR-type immune receptors. A characteristic hallmark of ETI is the hypersensitive response (HR), which is a form of rapid, localized cell death at the infection site. Based on genome and transcriptome analysis, large numbers of putative effectors have been predicted in the cyst nematodes, *Globodera spp*. and *Heterodera spp*. [3–5] and several of them have been demonstrated to be able to suppress ETI and/or PTI signaling [6–11]. For example, the *G*. *rostochiensis* effector, *GrUBCEP12* is processed into a free ubiquitin (Ub) and a short carboxyl extension peptide (CEP12) in plant cells [6]. CEP12, in turn, interferes with PTI signaling, as manifested by blockage of flg22-induced ROS production and PTI marker gene expression [7], and disruption of ETI signaling, as shown by suppression of HR mediated by the NB-LRR receptor Gpa2 [6].

The ubiquitin proteasome system plays a significant role in many plant physiological processes by removal of intracellular proteins. The ubiquitin pathway involves sequential action of E1 (ubiquitin activating enzyme), E2 (ubiquitin conjugating enzyme) and E3 (ubiquitin ligase) enzymes to covalently link ubiquitin to E3-specified substrate proteins which are then transported to the proteasome for degradation [12]. A growing body of evidence has suggested that pathogens may use effectors to manipulate host ubiquitin pathway to promote pathogenesis, during which effectors may either hijack the host ubiquitin system or themselves possess E3 activity to ubiquitinate host defense-related factors for degradation [13,14]. Significantly, few phytobacterial effectors have been demonstrated to act an E3 ubiquitin ligase for such virulent activity, including *Pseudomonas* effector AvrPtoB [15,16], *Xanthomonas* effector XopL [17], and *Ralstonia* effectors RipAW and RipAR [18]. However, no effectors have been identified as ubiquitin ligase from eukaryotic (plant or animal) pathogens, including fungi, oomycetes, or nematodes.

Although several plant-parasitic nematode effectors have been demonstrated to suppress PTI and ETI signaling, the mechanistic basis by which effectors manipulate host defense is unknown, mainly due to lack of understanding of the biochemical characteristics of the effectors. In this study, we identified a novel *G*. *pallida* RHA1B effector that is an E3 ubiquitin ligase, which resembles a typical *Really Interesting New Gene* (RING)-type E3 and can utilize multiple host E2 ubiquitin conjugation enzymes to catalyze ubiquitination. We demonstrated that RHA1B not only manipulates the host ubiquitin system as a functional E3 ubiquitin ligase to suppress ETI signaling, but also utilizes an E3 activity-independent mechanism to block PTI signaling.

## Results

### Characterization of RHA1B effector

**Fig 1:**
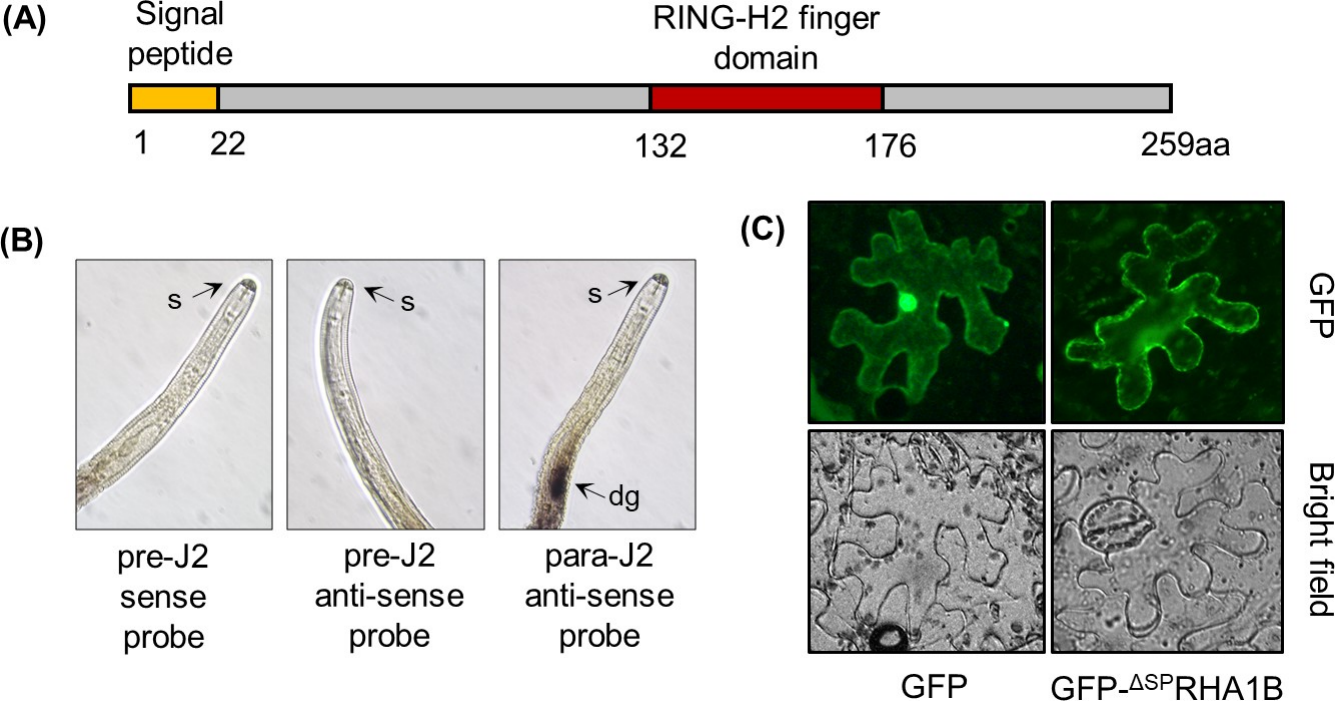
Expression of the *RHA1B* gene in *G. pallida* and localization of the RHA1B protein in plant cells. (A) Diagram of the RHA1B protein indicating the signal peptide and RING-H2 domains. (B) *RHA1B* is expressed in the dorsal esophageal gland. *In situ* hybridization of sections of *G. pallida* second-stage juveniles (J2) with the antisense probe binding to the dorsal gland cells of para-J2s. No signal was observed in pre-J2s incubated with the sense and anti-sense probes. dg–dorsal gland, s–stylet. (C) Subcellular localization of free GFP and GFP- ^ΔSP^RHA1B fusion protein in *N. benthamiana* leaves 36 hours after agro-infiltration. (B, C) Experiments were repeated three times with similar results.

The analysis of a *G*. *pallida* life stage-specific transcriptome revealed a long list of genes encoding potential effector proteins of unknown function [5]. The RHA1B (GPLIN_000167300), which was strongly induced (about six hundred eighty-fold) during the early parasitic stage [5], possessed a unique RING-H2 finger domain (C4H2C3-type) predicted by PROSITE [19] (Fig 1A) and contained an N-terminal signal peptide (SP) of 22 amino acids (as predicted by SignalP [20]). Since plant-parasitic nematode effectors are predominantly expressed in esophageal glands that are connected to a hollow protrusible stylet [21], the spatial expression of the *RHA1B* gene was determined by *in situ* hybridization. Consistent with the prediction of the dorsal gland-specific promoter element motif (DOG box) in the promoter of *RHA1B* gene [22], the digoxigenin (DIG)-labeled anti-sense cDNA probe of *RHA1B* exclusively hybridized to the dorsal esophageal gland cells in parasitic J2 (para-J2) nematodes (Fig 1B), whereas no signal was detected in pre-parasitic J2 (pre-J2) nematodes, suggesting that the expression of *RHA1B* gene is induced upon entrance of *G*. *pallida* into host roots.

Next we sought to test where RHA1B localizes in plant cells. Due to lack of RHA1B antibody that could be used to determine the localization of RHA1B in plant cells, we generated a N-terminal GFP-RHA1B fusion construct to examine the localization of RHA1B in plant cells as a GFP-RHA1B fusion protein. The GFP-fused RHA1B effector was transiently expressed in *Nicotiana benthamiana* leaves and visualized in epidermal leaf tissues by fluorescence microscopy. It is believed that the mature effector proteins lack the N-terminal SP, thus we used the RHA1B construct without SP in our study (referred as ^ΔSP^RHA1B hereafter). As shown in Fig 1C, the fluorescence signal of the CaMV 35S promoter-driven GFP-^ΔSP^RHA1B fusion protein was found exclusively in the cytoplasm, whereas the free GFP signal was present in both cytoplasm and nucleus, suggesting RHA1B does not localizes to any specific subcellular compartment when ectopically expressed as a GFP-fusion protein in *N*. *benthamiana* leaves.

### RHA1B is an E3 ubiquitin ligase

**Fig 2:**
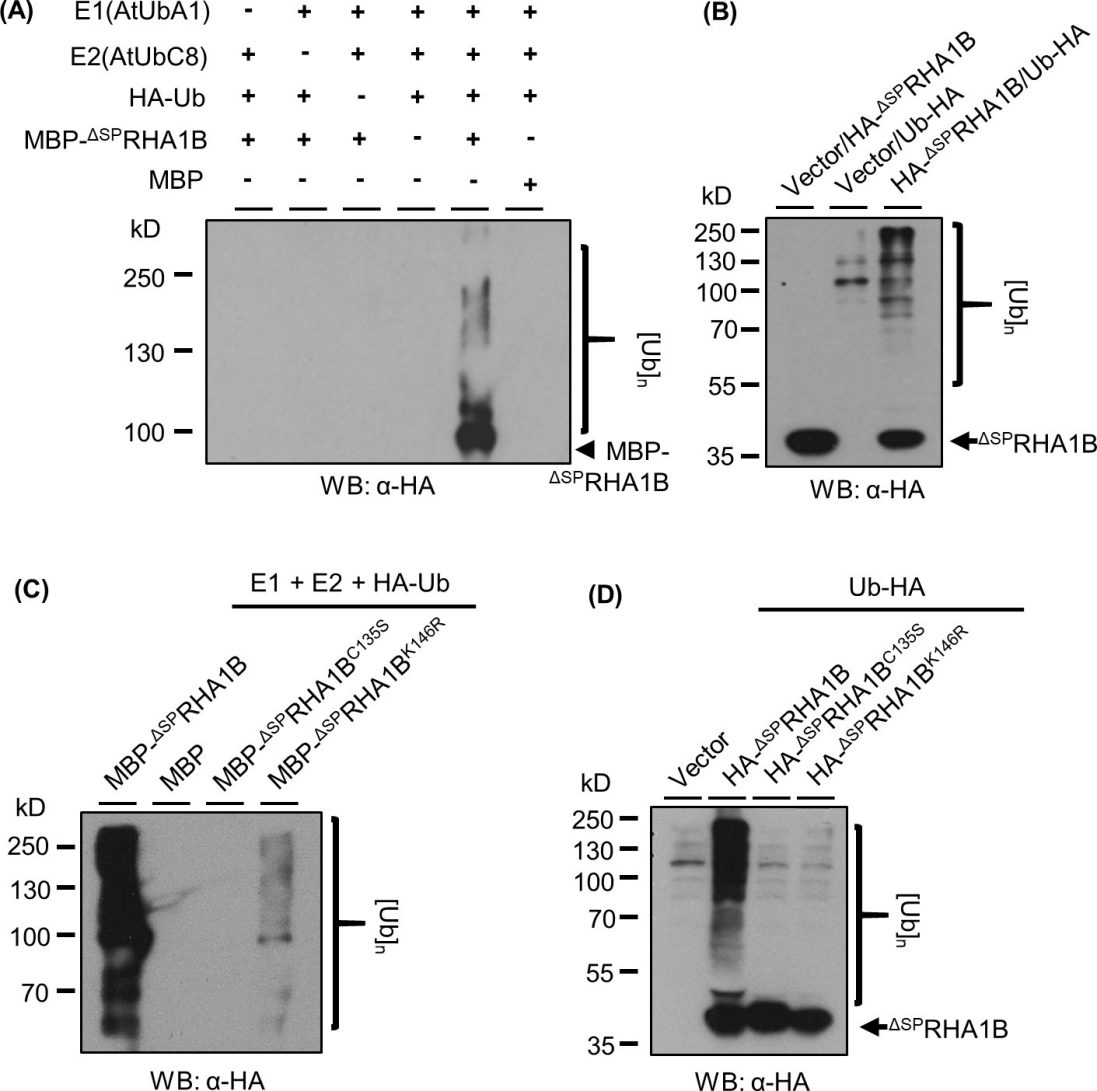
RHA1B is an active E3 ubiquitin ligase. (A) *in vitro* self-ubiquitination of RHA1B. Self-polyubiquitination of RHA1B was determined by Western blotting (WB) using anti-HA antibody. (B) RHA1B enhances ubiquitination *in planta*. Ubiquitination level in agro-infiltrated *N. benthamiana* leaves was determined through WB using anti-HA antibody. (C/D) Zn-finger motif and the Lys135 residue are essential for the intrinsic E3 activity of RHA1B. Similar *in vitro* ubiquitination (C) and *in vivo* ubiquitination assay (D) including the ^ΔSP^RHA1B^C135S^ and ^ΔSP^RHA1B^K146R^ mutants were conducted as in (A) and (B), respectively. (A-D) Experiments were repeated at least three times with similar results.

The amino acid sequence of RHA1B indicates that it could be a RING-type ubiquitin ligase possessing a typical C4H2C3 amino acid motif. To determine whether RHA1B has *in vitro* ubiquitination capability in the presence of ubiquitin enzymes E1 and E2 together with free ubiquitin [12], we incubated the recombinant MBP-tagged ^ΔSP^RHA1B (MBP-^ΔSP^RHA1B) protein with HA-tagged ubiquitin (HA-Ub) in the presence of ubiquitin E1 (*At*UbA1) and E2 (*At*UbC8), followed by Western blotting (WB) analysis using an anti-HA antibody. As shown in Fig 2A, when all components were present, a multi-banding smear began at the molecular weight position of MBP-^ΔSP^RHA1B and progressed upwards. This result, characteristic of self-ubiquitination with poly-ubiquitin, was abolished in various controls lacking either one or more components, suggesting RHA1B possesses E3 ligase activity. This enzymatic activity was further verified by the detection of RHA1B-promoted *in vivo* ubiquitination in plant cells. Although the substrates of RHA1B remain unidentified, transient co-expression of ^ΔSP^RHA1B and ubiquitin in *N*. *benthamiana* leaves resulted in a dramatically increased poly-ubiquitination signal in plant cells (Fig 2B lane 3), indicating the cellular event of ubiquitination was enhanced in the presence of ^ΔSP^RHA1B. Thus, we concluded that the *G*. *pallida* RHA1B effector is a functional E3 ubiquitin ligase.

### Amino acid residues essential for the ligase activity of RHA1B

The RING domain, typically consisting of two Zn-finger motifs stabilized by conserved Cys residues, is indispensable for the E3 ubiquitin ligase activity since it is involved in binding to the E2 conjugating enzyme in order to facilitate transfer of ubiquitin to a substrate protein. To determine whether the RING domain of RHA1B is required for its E3 activity, we generated a ^ΔSP^RHA1B^C135S^ mutant, in which the conserved Cys in the RING domain was substituted with a Ser. The *in vitro* ubiquitination assay indicated that the ^ΔSP^RHA1B^C135S^ mutant no longer has E3 activity (Fig 2C lane 3), suggesting that the Zn-finger motif is essential for the E3 activity. Beside the RING domain, Lys residue(s), which may serve as self-ubiquitination sites of E3, could also be important for the activity of an E3 ligase. Thus, we next determined whether replacing the Lys residue of RHA1B has an effect on its E3 activity. There is only one Lys residue (K146) in the mature ^ΔSP^RHA1B protein and we substituted it with Arg. When the ^ΔSP^RHA1B^K146R^ mutant was tested in the *in vitro* ubiquitination assay, only marginal poly-ubiquitination was observed (Fig 2C lane 4). This result suggested that this Lys is required either for the E3 activity of RHA1B or, as a ubiquitination site, for self-ubiquitination. To further clarify these possibilities, we conducted an *in vivo* ubiquitination investigation on the ^ΔSP^RHA1B^K146R^ mutant, with the wild type (WT) ^ΔSP^RHA1B and ^ΔSP^RHA1B^C135S^ mutant serving as positive and negative controls, respectively. As shown in Fig 2D, neither the ^ΔSP^RHA1B^K146R^ nor the ^ΔSP^RHA1B^C135S^ mutant could promote *in vivo* ubiquitination, suggesting that the E3 activity is abolished in the ^ΔSP^RHA1B^K146R^ mutant. Taken together, our results indicated that both the conserved Zn-finger motif and the Lys146 residue are indispensable for the intrinsic E3 activity of RHA1B.

### The plant host E2 specificity of RHA1B in catalyzing ubiquitination

**Fig 3:**
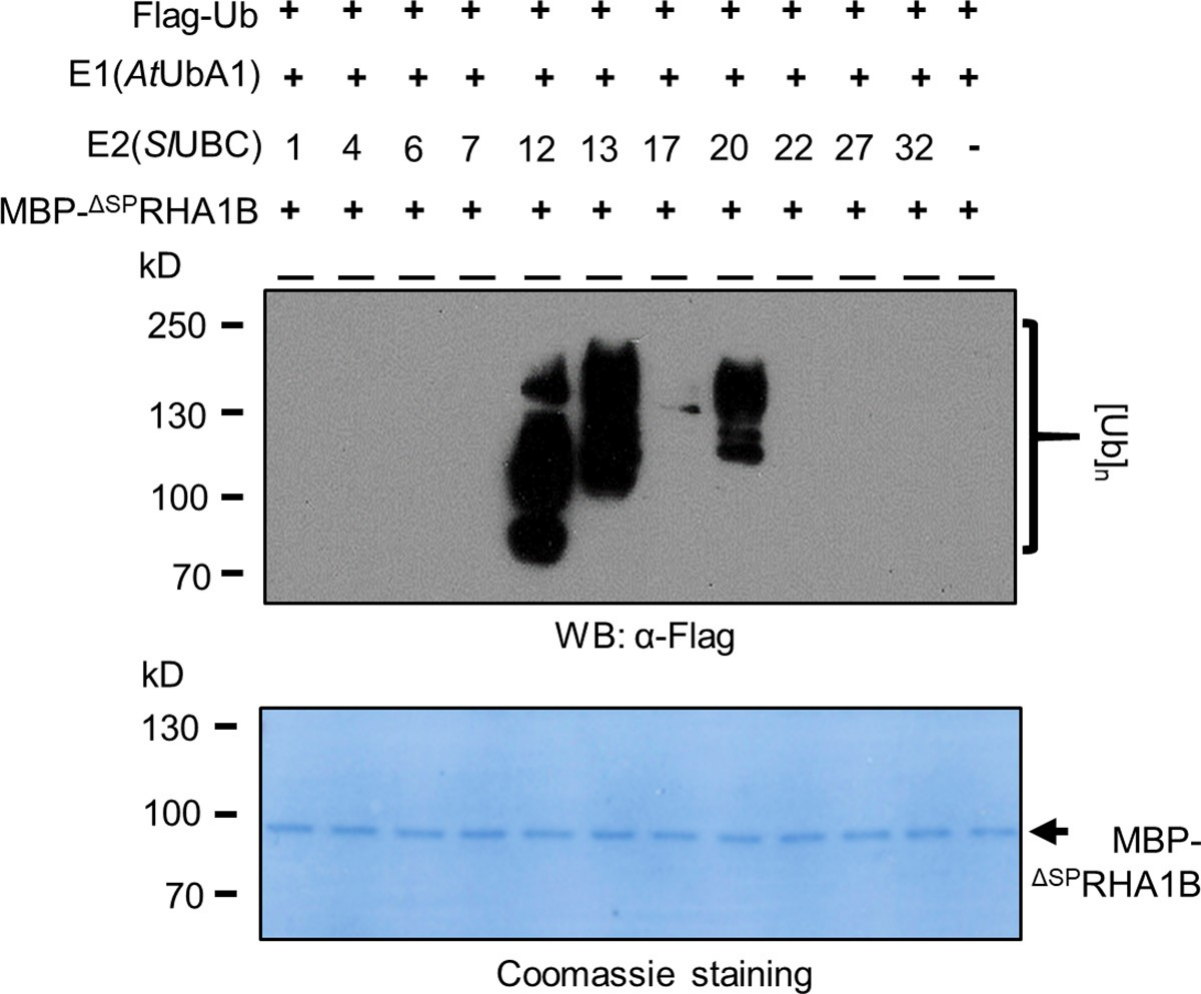
The E2 specificity of RHA1B ubiquitin ligases. *In vitro* ubiquitination assay with 10 different tomato E2 (SlUBC1/4/6/7/12/13/17/20/22/27/32) in the presence of MBP-^ΔSP^RHA1B, E1 and free Flag-Ub. Poly-ubiquitination indicated by the presence of smear banding was detected by WB using the anti-Flag antibody. The experiment was repeated three times.

The ubiquitination reaction mediated by the RING-type E3 ubiquitin ligase is accomplished by transferring ubiquitin to a substrate protein from an intermediate formed by the E2 ubiquitin-conjugating enzyme [12]. Many studies have shown the appropriate combination of E2 with E3 is critical for the potential activity of a given E3 [12]. Significantly, there are dozens of E2 ubiquitin conjugating enzymes in plants and each one may be involved in different cellular processes, thus determination of the E2 specificity of RHA1B would not only indicate which plant E2(s) could be exploited by RHA1B but also imply what host signaling pathways might be altered by RHA1B. Since tomato is a natural host of *G*. *pallida* and all tomato E2s have been characterized recently [23], we sought to determine the E2-E3 specificity between tomato E2 enzymes and RHA1B to define the E2 protein(s) that cooperates with RHA1B. To this end, one representative E2 (*Sl*UBC1/4/6/7/12/13/17/20/22/27/32) was randomly selected from 10 (II, VI, V, III, IX, X, VIII, XII, I, IV, respectively) tomato ubiquitin E2 families. Our *in vitro* ubiquitination assay indicated that, among these 10 E2s, *Sl*UBC12, 13 and 20 were able to facilitate the ubiquitination mediated by RHA1B (Fig 3), suggesting that RHA1B can exploit multiple E2 conjugating enzymes to potentially alter manipulate various cellular processes in host cells.

### RHA1B compromises flg22-triggered PTI signaling in an E3-independent manner

**Fig 4:**
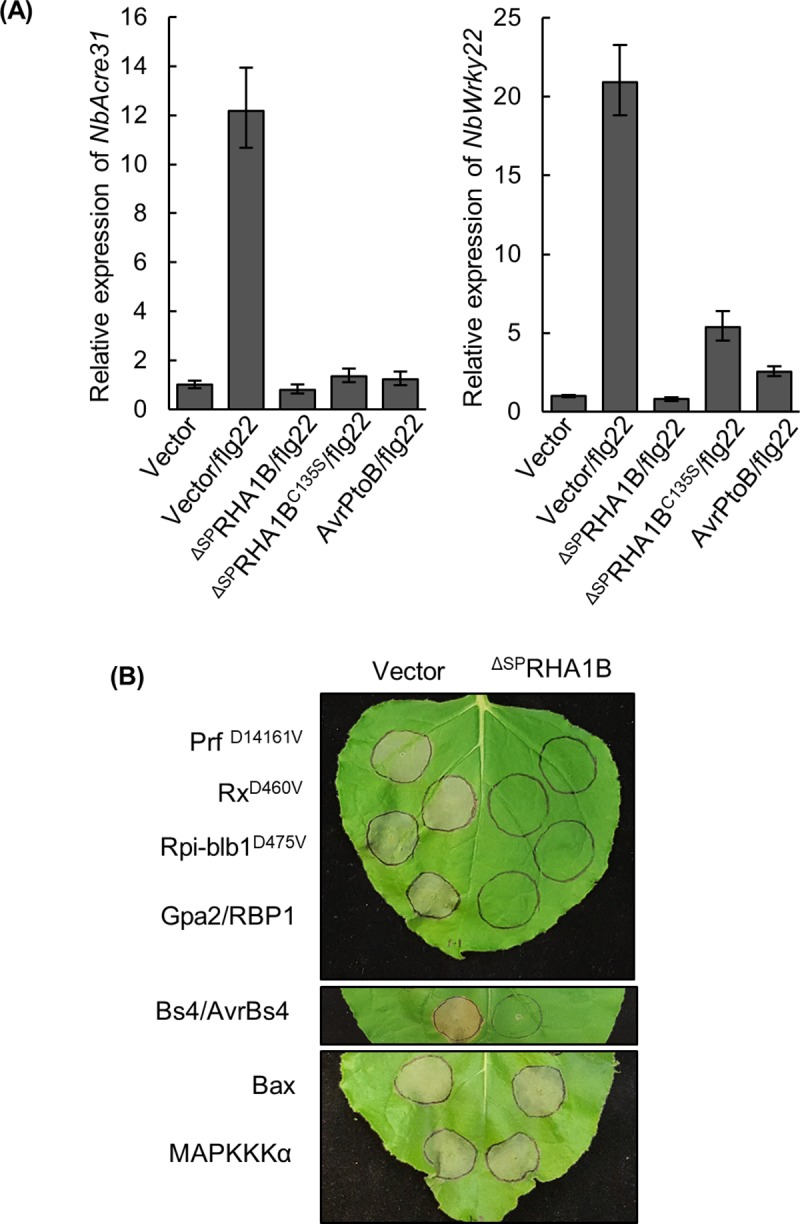
RHA1B suppresses PTI and ETI signaling in plants. (A) RHA1B suppresses expression of two PTI marker genes *NbAcre31* and *NbWRKY22* induced by flg22 in an E3-independent manner. The relative gene expression of *NbAcre31* and *NbWRKY22* was measured by qRT-PCR at one hour after flg22 treatment. The *NbEF1* gene was used as internal reference gene. Error bars are s.e.m., n = 3 biological replicates. (B) RHA1B suppresses HR mediated by a broad range of NB-LRR immune receptors. *N. benthamiana* leaves were agro-infiltrated with combination of either ^ΔSP^RHA1B or empty vector with auto-active NB-LRR mutant (Prf^D1416V^, Rx1^D460V^ or Rpi-blb1^D475V^), NB-LRR receptor and its cognate avirulence effector (Gpa2/RBP1 or Bs4/AvrBs4), or the pro-apoptotic mouse protein Bax and MAPKKKα at appropriate inoculum. Photographs were taken 5 days after agro-injection. (A, B) Experiments was repeated at least three times with similar results.

Next, we sought to determine the role of RHA1B in manipulating host defense to promote *G*. *pallida* parasitism. PTI activates the initial plant defense response to pathogen invasion and appears to be conserved across the plant kingdom. We adopted a well-established PTI signaling system triggered by the bacterial PAMP flg22 in *N*. *benthamiana* to determine whether RHA1B interferes with generic basal defense [24]. ^ΔSP^RHA1B was transiently expressed via *Agrobacterium* in *N*. *benthamiana* leaves for 24 hours. Parallel expression of vector or *Pseudomonas* effector AvrPtoB provided negative and positive control for PTI suppression, respectively. Duplicate agro-infiltrated areas were injected with 100nM flg22 to trigger PTI signaling. Tissue samples were collected one hour after flg22 treatment and assayed by quantitative Real-Time PCR (qRT-PCR) for the induction of two PTI marker genes, *NbAcre31* and *NbWRKY22* [25]. Our results indicated that, like the phytobacterial effector AvrPtoB, RHA1B blocked flg22-induced PTI signaling (Fig 4A), suggesting that RHA1B is capable of interfering with host basal defenses during nematode infection. In addition, we determined the role of the E3 activity in RHA1B-mediated suppression on PTI by examining the effect of the E3-deficient ^ΔSP^RHA1B^C135S^ mutant on PTI signaling. ^ΔSP^RHA1B^C135S^ was included in a similar *Agrobacterium*-mediated transient assay on flg22-triggered *NbAcre31* and *NbWRKY22* expression. Our results indicated that ^ΔSP^RHA1B^C135S^ still blocks *NbAcre31* and *NbWRKY22* induction by flg22 (Fig 4A), suggesting that the E3 activity of RHA1B is dispensable for interference with PTI signaling.

### RHA1B suppresses HR cell death mediated by a broad range of NB-LRR receptors

The HR cell death developed on *N*. *benthamiana* leaves, resulting from *Agrobacterium*-mediated transient co-expression of a plant NB-LRR protein and its cognate pathogen avirulence effector or expression of an auto-active NB-LRR mutant, has been widely considered as a diagnostic indicator of ETI. Thus, we used this fast and reliable system to determine whether RHA1B can interfere with the ETI signaling. We first tested the NB-LRR receptor Gpa2 that confers resistance to *G*. *pallida* in potato and our result showed that RHA1B can suppress cell death triggered by co-expression of Gpa2 and RBP-1 [26] (Fig 4B). Since Gpa2 belongs to the CC-NB-LRR subgroup (containing a coiled-coil (CC) domain at its N terminus) of NB-LRR receptor and confers resistance to nematode, we asked whether or not the cell death suppression activity of RHA1B is specific to Gpa2-mediated ETI signaling. We included in our cell death suppression assay other NB-LRR receptors, which confer resistance to bacterium, virus or oomycete and belong to CC-NB-LRR or NTR-NB-LRR (containing a Toll-interleukin 1-like receptor (TIR) domain at its N terminus) subgroup. The tested NB-LRR receptors were Rx1 (conferring resistance to virus, CC-NB-LRR subgroup) [27], Prf (conferring resistance to the bacterium, CC-NB-LRR subgroup) [28], Rpi-blb1 (conferring resistance to the oomycete, CC-NB-LRR subgroup) [29], Bs4 (conferring resistance to bacterium; NTR-NB-LRR subgroup) [30]. As shown in Fig 4B, RHA1B was able to suppress cell death mediated by these NB-LRR immune receptors, where cell death was initiated by the auto-active NB-LRR mutant alone or the WT NB-LRR with its cognate avirulent effector. However, RHA1B was unable to block cell death induced by the pro-apoptotic mouse protein Bax [31] or MAPKKKα that is a downstream component of ETI signaling [32]. Taken together, our results not only indicated RHA1B can suppress ETI signaling mediated by a broad range of NB-LRR receptors, but also suggested that RHA1B is not a general cell death suppressor and it may target the early step(s) of ETI signaling.

### RHA1B requires its intrinsic E3 activity to trigger degradation of NB-LRR receptors, thereby suppressing ETI signaling

**Fig 5:**
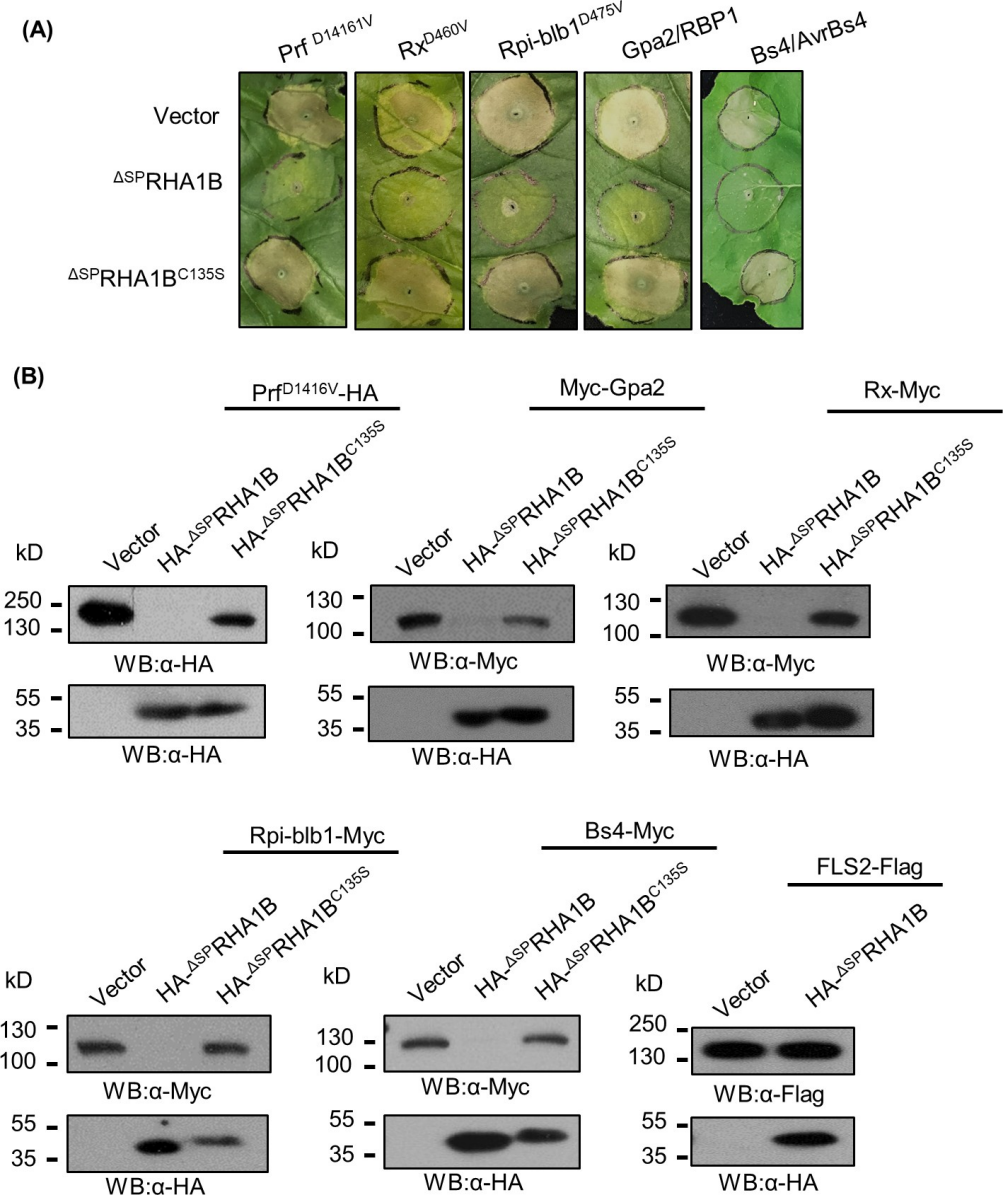
RHA1B suppresses ETI signaling by promoting the E3-dependent degradation of the NB-LRR receptor. (A) The E3 ubiquitin ligase activity of RHA1B is required for HR cell death suppression. *N. benthamiana* leaves were agro-infiltrated with combination of either ^ΔSP^RHA1B, empty vector, or ^ΔSP^RHA1B^C135S^ with NB-LRR receptor and its cognate avirulence effector (Gpa2/RBP1 or Bs4/AvrBs4) or with the auto-active NB-LRR mutant (Prf^D1416V^, Rx1^D460V^ or Rpi-blb1^D475V^) at appropriate inoculum. Photographs were taken 5 days after agro-injection. (B) RHA1B promotes degradation of NB-LRR immune receptors. The accumulation of NB-LRR or PRR proteins in the presence of ^ΔSP^RHA1B, empty vector, or ^ΔSP^RHA1B^C135S^ was determined by WB using appropriate antibodies. (A, B) Experiments were repeated at least three times with similar results.

We next sought to investigate molecular mechanism by which RHA1B suppresses ETI signaling. Given that RHA1B effector is an E3 ubiquitin ligase whose main function is to ubiquitinate proteins for proteasome-mediated degradation, it was logical to hypothesize that RHA1B relies on its E3 activity to specifically promote degradation of NB-LRR receptors, thereby suppressing ETI signaling. To test this notion, we first examined the cell death suppression capability of the E3-deficient ^ΔSP^RHA1B^C135S^ mutant. Our result showed that the ^ΔSP^RHA1B^C135S^ mutant cannot suppress cell death mediated by any of the tested NB-LRR receptors (Fig 5A). Next, we determined whether RHA1B does indeed trigger degradation of NB-LRR receptors in plant cells. It is notable that the auto-active Rx1^D461V^ and Rpi-blb1^D475V^ trigger extremely strong cell death in *N*. *benthamiana* accompanied by non-specific protein degradation that makes Rx1^D461V^ and Rpi-blb1^D475V^ proteins undetectable by Western blotting [33], thus we used the WT Rx1 and Rpi-blb1 in our assay. We co-expressed the epitope-tagged WT or auto-active NB-LRR receptors Myc-Gpa2, Prf^D1416V^-HA, Myc-Rx1, Rpi-blb1-HA, or Bs4-Myc with HA-^ΔSP^RHA1B, HA-^ΔSP^RHA1B^C135S^ mutant, or empty vector in *N*. *benthamiana* leaves and monitored the protein levels of those NB-LRR receptors. Our Western blotting analysis indicated that all tested NB-LRR receptors fail to accumulate in the presence of RHA1B. Significantly, such RHA1B-promoted degradation of NB-LRR receptors was completely dependent on the E3 activity of RHA1B as all tested NB-LRR receptors accumulated normally when co-expressed with the E3-deficient HA-^ΔSP^RHA1B^C135S^ mutant (Fig 5B).

Since the E3 activity of RHA1B was dispensable for interference with PTI signaling (Fig 4A), our results also suggest that RHA1B possesses two distinct activities to suppress ETI and PTI, one E3-dependent and one E3-independent, respectively. To verify the indispensability of E3 activity on PTI suppression, we examined whether ^ΔSP^RHA1B is able to promote degradation of FLS2, the PRR receptor for the PAMP flg22 [34]. ^ΔSP^RHA1B did not trigger degradation of FLS2 (Fig 5B), further indicating that RHA1B targets unknown component(s) other than PRR receptor of the PTI signaling pathway.

### Overexpression of RHA1B in transgenic potato enhanced the host susceptibility to *G*. *pallida*

**Fig 6:**
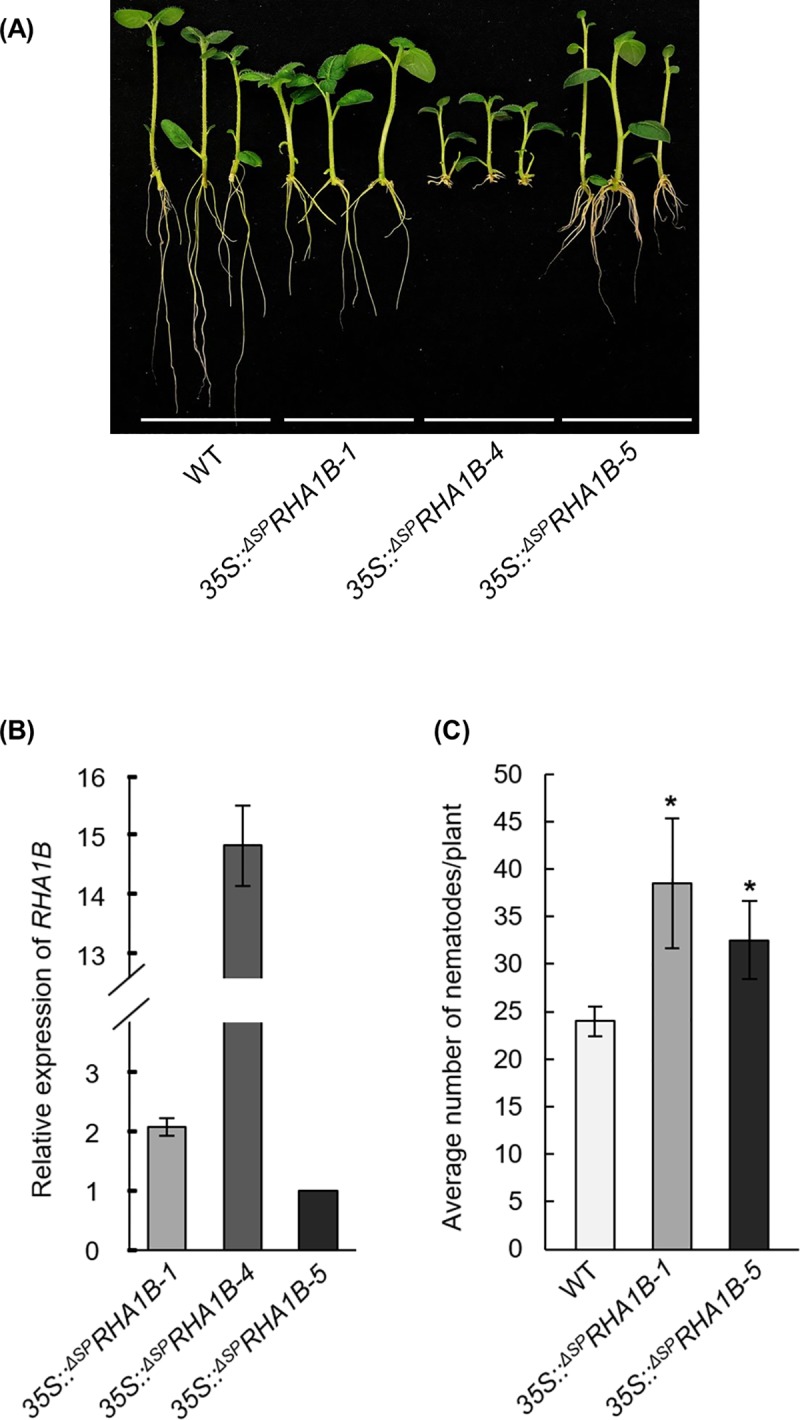
Over-expression of RHA1B in potato enhances the susceptibility to *G. pallida*. (A) Overexpression of *RHA1B* in transgenic potato results in root growth defect. (B) The relative expression of the *RHA1B* transgene in transgenic potato lines measured by qRT-PCR. The transcript value of *35S:: ^ΔSP^RHA1B-5* was normalized to 1. The potato *StActin* gene was used as internal reference gene. Error bars are s.e.m., n = 3 biological replicates. (C) RHA1B enhances potato susceptibility to *G. pallida*. Multiple vegetatively propagated plantlets of transgenic lines *35S:: ^ΔSP^RHA1B-1* and *-5* or wild-type (WT) Desiree control plants were inoculated with *G. pallida* and all nematodes were counted 6 weeks after plant inoculation. Error bars are s.d., n = 10 biological replicates. Statistical significance was determined by a two-tailed, unpaired Student’s t-test. *P < 0.05. The experiment was repeated two times with similar results.

To obtain genetic evidence for an involvement of the RHA1B in nematode parasitism, we sought to generate transgenic potato plants overexpressing *RHA1B* under the constitutive cauliflower mosaic virus (CaMV) 35S promoter and determine the altered susceptibility to *G*. *pallida*. Given the fact that RHA1B is a strong ubiquitin ligase that might target many host proteins for ubiquitination and degradation, which could affect various physiological processes of the host cells, it is possible that overexpression of *RHA1B* results in development and/or growth defects in potato plants. Even though much effort has been spent to generate transgenic potato plants expressing *35S*::^Δ*SP*^*RHA1B* construct, only three transgenic lines (designated as *35S*::^Δ*SP*^*RHA1B-1*, *-4*, *-5*, respectively) have been obtained. Significantly, all transgenic potato plants exhibited different levels of inhibition on root growth (Fig 6A). qRT-PCR assay on root tissues indicated that *35S*::^Δ*SP*^*RHA1B-4* expressed ^Δ*SP*^*RHA1B* at extreme high level (about 15 fold higher than line *35S*::^Δ*SP*^*RHA1B-5*), whereas *35S*::^Δ*SP*^*RHA1B-1* and *-5* exhibited moderate levels of ^Δ*SP*^*RHA1B* expression (Fig 6B). In consistence with the qRT- PCR results, the root growth of *35S*::^Δ*SP*^*RHA1B-4* plants was almost completely arrested (Fig 6A), therefore, this line was not suitable for nematode infection assay. Nevertheless, *35S*::^Δ*SP*^*RHA1B-1* and *-5* transgenic lines were examined for their susceptibility to *G*. *pallida* infection. Although we cannot completely rule out the possibility that the enhanced number of nematodes in the transgenic potato roots compared to that in the WT potato roots (Fig 6C) is an artifact of the abnormal root development, our data strongly suggest that RHA1B plays a significant role in *G*. *pallida* parasitism.

## Discussion

### Suppression of PTI signaling by RHA1B is independent of its E3 activity

Cyst nematodes move along host roots intracellularly penetrating root cells where they likely trigger DAMP-induced defenses. In order to mount a successful infection, they need to manipulate host PTI signaling. Indeed, the cyst nematode effector *Ha*ANNEXIN and the effector-derived CEP12 peptide have been shown to compromise flg22-induced ROS production and expression of PTI marker genes *Pti5* and *Acre31* [7,8]. Based on the flg22-triggered PTI marker gene expression, we concluded that RHA1B also blocks flg22-induced PTI signaling in our experimental system (Fig 4A). Significantly, this PTI suppression is not dependent on the E3 activity of RHA1B, as the E3-deficient ^ΔSP^RHA1B^C135S^ mutant was still able to suppress the induction of *NbAcre31* or *NbWRKY22* by flg22 (Fig 4A), further supporting that RHA1B possesses a distinct E3-independent virulence activity. Similar E3-independent PTI-suppression activity has been found in the phytobacterial effector AvrPtoB. The N-terminus of ArvPtoB lacking E3 activity is able to suppress PTI signaling, whereas the C-terminus of AvroPtoB possesses and E3 activity responsible for ETI suppression [35,36]. Thus, we speculate RHA1B, like the phytobacterial effector AvrPtoB, possesses two distinct virulence potentials (one is responsible for PTI suppression and the other one interferes with ETI (discussed below)) that involve different virulence‐promoting mechanisms.

### RHA1B suppresses HR cell death mediated by a broad range of NB-LRR receptors

After its mobile phase, a cyst nematode commits itself to form a feeding site (syncytium) in host roots and becomes sessile for the rest of its life. Thus, protection of the feeding site, which is the only source of food available for nematode to achieve its reproductive stage, is essential. The potato NB-LRR receptor Gpa2 specifically limits *G*. *pallida* infection by recognizing the nematode *Gp*RBP1 effector to trigger HR cell death. These dead cells can form a barrier to separate the syncytium from cells in the vascular bundle that provide the nutrients for nematode [1]. Therefore, *G*. *pallida* must suppress such HR cell death-dependent host immunity for survival. Recently a few cyst nematode effectors have been shown to suppress the HR cell death mediated by NB-LRR immune receptors. These effectors include *Gr*UBCEP12[6], *Ha*ANNEXIN [8], *Gr*EXPB2 [9], and SPRYSEC family members unique to the genus *Globodera* (*Gr*SPRYSEC-19 [11], *Gr*SPRYSEC-4, *Gr*SPRYSEC-5 and *Gr*SPRYSEC-8, *Gr*SPRYSEC-18 [10]). Interestingly, *Gr*SPRYSEC19 and *Gr*UBCEP12 can suppress cell death mediated by Gpa2 that provides resistance to some isolates of *G*. *pallida* [6,11]. We found *G*. *pallida* effector RHA1B can suppress HR cell death mediated by a broad range of NB-LRR immune receptors, including the CC-NB-LRR subgroup Gpa2, Prf, Rpi-blb1, Rx1, and the TIR-NB-LRR subgroup Bs4 (Fig 4B). Although only Gpa2 is related to nematode resistance, our data suggest *G*. *pallida* has evolved RHA1B as a powerful effector with potential to subvert host immunity mediated by a broad range of NB-LRR receptors. However, RHA1B was unable to block cell death triggered by the pro-apoptotic mouse protein Bax or the HR cell death signaling component MAPKKKα (Fig 4B), suggesting that RHA1B does not act as a general cell death suppressor. This phenomenon is consistent with our further analysis showing that RHA1B-dependent cell death suppression is correlated with RHA1B-triggered specific degradation of the NB-LRR receptors.

We have also examined possible *in planta* interaction between RHA1B with Gpa2 by co-immunoprecipitation assay. Our results indicated that RHA1B does not interact with Gpa2 *in vivo* (S1 Fig), suggesting that RHA1B does not directly ubiquitinate Gpa2 for degradation. Nevertheless, given that the intrinsic E3 activity of RHA1B is required for both degradation of NB-LRR receptors and HR cell death suppression, we hypothesize that RHA1B indirectly targets NB-LRR receptors for degradation. For example, RHA1B might ubiquitinate and promote proteolysis of as yet unidentified factor(s), such as molecular chaperones and/or co-chaperones that help fold or stabilize NB-LRR proteins.

### Manipulation of host ubiquitin pathway by RHA1B as a virulence mechanism

A growing body of evidence has shown that the ubiquitin pathway plays important role in the plant-nematode interactions. Several nematode effectors have been described as potential components of the ubiquitin pathway. For example, *Gr*UBCEP12 is a ubiquitin carboxyl extension protein that is processed into a free ubiquitin and CEP12 in plant cells [6]; *Gr*SKP1 is an E3 adaptor-like protein [9,37]. However, these nematode effectors differ from RHA1B in that they likely rely on exploiting host E3 ligases, rather than embodying such enzymatic activity. RHA1B is a nematode effector equipped with E3 ligase activity, by which it promotes degradation of NB-LRR receptors to interfere with ETI signaling. To our knowledge, this is the first effector with E3 ubiquitin ligase identified from eukaryotic (plant or animal) pathogens, including fungi, oomycetes and nematodes.

Although the E3 ligase confers the substrate specificity of ubiquitination, the E2-E3 combination cooperatively determines the topology of the polyubiquitin chain, which determines the fate of the ubiquitinated protein [38]. We found that RHA1B can function in concert with three host E2s (*Sl*UBC12, 13 and 20) when ubiquitinating *in vitro* (Fig 3). Among these three E2s, *Sl*UBC13 exclusively catalyzes the Lys-63-linked ubiquitination [39], which is generally involved in non-proteolytic processes, such as protein trafficking, translation and DNA repair [40]. In contrast, *Sl*UBC12 belongs to group III of the tomato E2s that are also used by the phytobacterial effector AvrPtoB to ubiquitinate host defense-related proteins for degradation [23]. It is also notable that three E2s possess distinct ubiquitination potential when catalyzing ubiquitination with RHA1B *in vitro*, as indicated by difference in the size range of ubiquitination-associated smears observed in the *in vitro* ubiquitination assay (Fig 3). The ability of RHA1B to manipulate host plant physiological processes is further supported by the phenotypic changes observed in transgenic potato plants over-expressing RHA1B. All transgenic potato plants exhibited different levels of arrest on root development and growth, which was correlated with the expression levels of the *RHA1B* transgene (Fig 6A & 6B). Moreover, the tested *35S*::^Δ*SP*^*RHA1B-1* and *-5* transgenic lines exhibited enhanced susceptibility to *G*. *pallida* infection (Fig 6C), despite the fact that we cannot completely rule out the possibility that the enhanced numbers of nematodes in the transgenic potato roots could be an artifact of the abnormal root development caused by over-expression of the RHA1B effector. Thus, we hypothesize that the ability of RHA1B to exploit multiple host E2s could arm *G*. *pallida* with a unique advantage in parasitism in which the RHA1B-E2 combinations provide the parasite with strategy to affect a wider range of physiological processes of host plants via manipulation of both proteolytic and non-proteolytic protein process in infected hosts.

## Materials and methods

### Generation of constructs

The *RHA1B* gene without signal peptide-coding region was PCR-amplified from the *G*. *pallida* J3 cDNA using primers listed in S1 Table. *RHA1B* was cloned into pBIN-ARS vector [41] carrying 5’-terminal sequences encoding GFP or HA epitope tags for *Agrobacterium*-mediated transient expression or generation of transgenic potato plants, respectively. The C135S or K146R substitution in RHA1B was introduced using PfuUltra (Agilent, Santa Clara, CA, USA) polymerase-driven PCR, the resulting ^ΔSP^RHA1B^C135S^ and ^ΔSP^RHA1B^K146R^ mutants were verified by DNA sequencing and cloned into the pMAL-c2 vector (NEB, Ipswich, MA, USA) to generate recombinant protein.

### *In situ* hybridization

In situ hybridizations were performed using preparasitic J2s (pre-J2s) and parasitic J2s (para-J2s) of G. *pallida* nematodes isolated from inoculated *Desiree* potato plants following the protocol described in a previous publication [42]. The DNA probes specific for the digoxigenin (DIG)-labelled sense (negative control) and antisense single-stranded cDNA were synthesized using a PCR DIG probe Synthesis Kit (Roche Applied Science, Indianapolis, USA). Hybridization signals within the nematodes were detected using alkaline phosphatase conjugated antidigoxigenin antibody (diluted 1:100) and substrate, and the nematode sections were observed using a stereo-microscope (Leica Microsystems, Wetzlar, Germany) to detect the hybridized probes in the nematodes tissue. Primers used for *in situ* hybridization are listed in S1 Table.

### *Agrobacterium*-mediated transient protein expression and western blotting

*Agrobacterium*-mediated transient expression on *N*. *benthamiana* leaves was carried out as described previously [35]. *Agrobacterium tumefaciens* GV2260 strains expressing differentially tagged proteins were syringe-infiltrated into *N*. *benthamiana* leaves. The concentrations of agrobacterial inoculum varied from OD_600_ = 0.05 to OD_600_ = 0.4. For the subcellular localization assay, *A*. *tumefaciens* GV2260 strain containing the appropriate GFP chimera construct was injected into *N*. *benthamiana* leaves. After 36 hours, the epidermal cell layers were examined using confocal microscope (Olympus) to capture the GFP signal.

For Western blotting assay, *Agrobacterium*-infected *N*. *benthamiana* leaf tissues were collected at 28–36 hours after infiltration and ground with liquid nitrogen. The fine tissue powder was resuspended with 300 μl of protein extraction buffer (50mM Tris-HCl pH 7.5, 150mM NaCl, 5mM EDTA, 2mM DTT, 10% glycerol, 1% polyvinylpolypyrolidone, 1mM PMSF, plant protease inhibitor cocktail (Sigma-Aldrich, Saint Louis, USA)) and centrifuged at 13,000g/4°C for 15 minutes. Protein samples were separated on 10% SDS-PAGE gels, transferred onto PVDF membrane and probed with anti-HA (Sigma-Aldrich, Saint Louis, USA, Cat# H3663; RRID:AB_262051), anti-FLAG (Sigma-Aldrich, Saint Louis, USA, Cat# F3165; RRID:AB_259529), or anti-Myc primary antibody (Sigma-Aldrich, Saint Louis, USA, Cat# M4439; RRID:AB_439694), followed by anti-mouse secondary antibody (Sigma-Aldrich, Saint Louis, USA, Cat# A9044; RRID:AB_258431). Protein signal was detected with ECL Prime (GE Healthcare, Chicago, USA).

### *In vitro* ubiquitination assay

The pMAL-c2 plasmid harboring MBP-RHA1B, MBP-RHA1B^C135S^ or MBP-RHA1B^K146R^ construct was expressed in *E*. *coli* BL21 using 0.5μM IPTG for induction. Recombinant proteins were purified using the Amylose Resin (NEB, Ipswich, USA) following the manufacturer’s instructions. The *in vitro* ubiquitination assay was performed as described previously [16] with few adjustments. 40ng His-E1 (*At*UBA1), 100ng His-E2 (*At*UBC8), 1μg MBP*-*RHA1B (or MBP-RHA1B^C135S^ or MBP-RHA1B^C135S^), 2μg HA-Ub (Boston Biochem, Boston, USA) were incubated in the ubiquitination buffer (50mM Tris HCl, pH7.5, 2mM ATP, 5mM MgCl_2_, 30mM creatine phosphate (Sigma-Aldrich, St. Louis, USA) containing 50ng/μl creatine phosphokinase (Sigma-Aldrich, St. Louis, USA)). The 30μl reaction mixture was incubated for 2 hours at 30°C. Proteins were separated on 7.5% SDS-PAGE gels and identified by Western blotting using anti-HA antibody (Sigma-Aldrich, St. Louis, USA).

### Flagellin treatment and quantitative real-time PCR (qRT-PCR)

*N*. *benthamiana* leaves were agroinfiltrated with desired constructs for transient protein expression. 36 hours after infiltration, either water control or 100nM flg22 (PhytoTechnology Laboratories, Shawnee Mission, USA) were injected to induce *NbAcer31* and *NbWRKY22* expression. Total RNA from agroinfiltrated leaf discs treated with flg22 for 1 hour was isolated with TRIzol reagent (Invitrogen, Carlsbad, USA). One microgram of total RNA was treated with DNase I (Invitrogen, Carlsbad, USA), followed by reverse transcription using a Super Script II reverse transcriptase (Invitrogen, Carlsbad, USA). qRT-PCR analysis was performed on an ABI Prism 7100 sequence detection system using Power SYBR Green reagents (Life Technologies, Carlsbad, USA). The *N*. *benthamiana EF1* gene was used as an internal control for normalization [43]. Relative expression ratios were determined based on the comparative CT method (ΔΔCT) using the StepOne Software. Primers used in qRT-PCR are listed in S1 Table.

### Nematode infection assay on transgenic potato plants over-expressing *RHA1B*

Transgenic potato plants over-expressing *RHA1B* were generated via *Agrobacterium*-mediated transformation [44]. Propagated plants of the transgenic lines were used for nematode inoculation. Transgenic and non-transgenic control plant seedlings were inoculated with 10 *G*. *pallida* cysts in the root zone for six weeks. All life stages of nematode were counted from individual plants using acid fuchsin assay [45].

## Supporting information

S1 Fig RHA1B does not interact with Gpa2 *in vivo*. Neither **(A)** ^ΔSP^RHA1B^C135S^ E3-ligase deficient mutant nor **(B)** ^ΔSP^RHA1B (in the presence of MG132 proteasomal inhibitor to prevent Gpa2 degradation) immunoprecipitated with Gpa2 *in vivo*. Immunoprecipitation was carried out with anti-HA agarose beads. The accumulation of tested proteins was verified by WB using appropriate antibodies.
(PDF)

S1 Table Primers used in this study. (PDF)

S1 File Supplemental method. (PDF)
