# Supplementary material for: The potato cyst nematode effector RHA1B is a ubiquitin ligase and uses two distinct mechanisms to suppress plant immune signaling
Source: PLoS Pathog. 2019 Apr 12;15(4):e1007720. doi: 10.1371/journal.ppat.1007720 (PMC6461251; doi:10.1371/journal.ppat.1007720)
Supplement: S1 Fig — Neither (A) ΔSPRHA1BC135S E3-ligase deficient mutant nor (B) ΔSPRHA1B (in the presence of MG132 proteasomal inhibitor to prevent Gpa2 degradation) immunoprecipitated with Gpa2 in vivo. Immunoprecipitation was carried out with anti-HA agarose beads. The accumulation of tested proteins was verified by WB using appropriate antibodies. (PDF) [file ppat.1007720.s001.pdf]

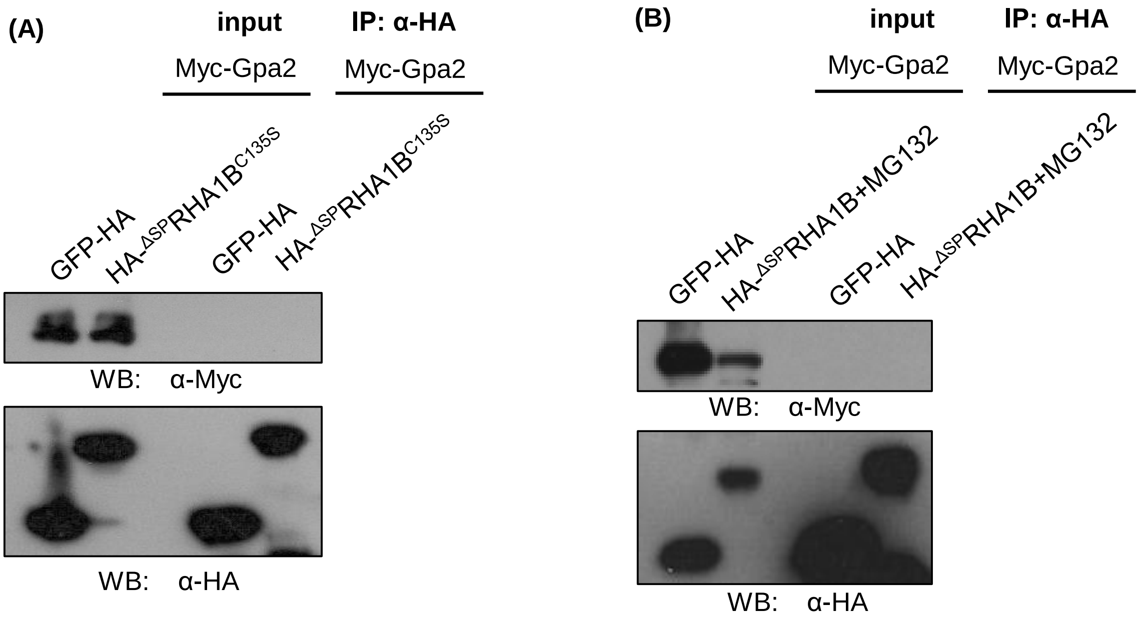

S1 Fig. RHA1B does not interact with Gpa2 *in vivo*. Neither **(A)** $^{\Delta SP}$RHA1B^{C135S} E3-ligase deficient mutant nor **(B)** RHA1B (in the presence of MG132 proteasomal inhibitor to prevent Gpa2 degradation) immunoprecipitated with Gpa2 *in vivo*. Immunoprecipitation was carried out with anti-HA agarose beads. The accumulation of tested proteins was verified by WB using appropriate antibodies.
