# Supplementary material for: The potato cyst nematode effector RHA1B is a ubiquitin ligase and uses two distinct mechanisms to suppress plant immune signaling
Source: PLoS Pathog. 2019 Apr 12;15(4):e1007720. doi: 10.1371/journal.ppat.1007720 (PMC6461251; doi:10.1371/journal.ppat.1007720)
Supplement: S1 Table — (PDF) [file ppat.1007720.s002.pdf]

S1 Table. Primers used in this study

Name	Sequence	Construct
RHA1B FBamHI ΔSP	agggatccggcaatttatttaacattggcggtaa	pBTEX::GFP-RHA1B<br>pBTEX::HA-RHA1B<br>pBIN::HA-RHA1B<br>pMAL-c2::MBP-RHA1B
RHA1B RSalI	aggtcgactcatggagactccggtgggc	
RHA1B C135S F	gcggagtgcccaattagtttggaatcattgtca	mutagenesis C135S
RHA1B C135S R	tgacaatgattccaaactaattgggcactccgc	
RHA1B K146R F	gcggaagcagatgtgagggaattgcgctcttgc	mutagenesis K146R
RHA1B K146R R	gcaagagcgcaattccctcacatctgcttccgc	
*Nb*EF1 RTF	aaggtccagtatgcctgggtgcttgac	qRT-PCR
*Nb*EF1 RTR	aagaattcacagggacagttccaatacca	
*Nb*Acre31 RTF	aattcggccatcgtgatcttggtc	
*Nb*Acre31 RTR	gagaaactgggattgcctgaagga	
*Nb*WRKY22 RTF	aaggtccgggatctacatgcggtggt	
*Nb*WRKY22 RTR	aagaattccgggtcggatctatttcg	
*St*Actin97 RTF	ctcttgactatgaacaggaac	
*St*Actin97 RTR	aaggacctcaggacaccg	
RHA1B RTF	tgcgaaccactcaactcctcc	
RHA1B RTR	cacatctgcttccgctgacaat	
RHA1B ISHF	tggcggtaattctggttctc	*in situ* hybridization
RHA1B ISHR	caaggttcggaggagttgag	
