# Supplementary material for: The potato cyst nematode effector RHA1B is a ubiquitin ligase and uses two distinct mechanisms to suppress plant immune signaling
Source: PLoS Pathog. 2019 Apr 12;15(4):e1007720. doi: 10.1371/journal.ppat.1007720 (PMC6461251; doi:10.1371/journal.ppat.1007720)
Supplement: S1 File — (PDF) [file ppat.1007720.s003.pdf]

S1 Method

Agrobacterium-infected *N. benthamiana* leaf tissues were collected at 32 hours after infiltration and ground with liquid nitrogen. The fine tissue powder was resuspended with 1 ml of protein extraction buffer (50mM Tris-HCl pH 7.5, 150mM NaCl, 5mM EDTA, 2mM DTT, 10% glycerol, 1% polyvinylpolypyrolidone, 1mM PMSF, plant protease inhibitor cocktail (Sigma-Aldrich, Saint Louis, USA)) and centrifuged at 13,000g/4°C for 20 minutes. One-tenth of each protein extract (v:v) was saved as the input sample, and the rest of the of the protein solution was used for precipitation with 15μl α-HA affinity matrix (Roche Applied Science, Indianapolis, IN, USA) for two hours at 4°C. The immunoprecipitated protein complex was washed four times with washing buffer (50mM Tris-HCl, pH 7.5, 250mM NaCl, 5mM EDTA, 10% glycerol, 1mM PMSF). Protein samples were boiled for 5min, separated on 10% SDS-PAGE gels, transferred onto PVDF membrane and probed with anti-HA or anti-Myc primary antibody, followed by anti-mouse secondary antibody (Sigma-Aldrich, Saint Louis, USA). Protein signal was detected with ECL Prime (GE Healthcare, Chicago, USA).
